# Development of a Clinical Decision Support System for Pediatric Abdominal Pain in Emergency Department Settings Across Two Health Systems Within the HCSRN

**DOI:** 10.5334/egems.282

**Published:** 2019-04-12

**Authors:** Heidi L. Ekstrom, Elyse O. Kharbanda, Dustin W. Ballard, David R. Vinson, Gabriela Vazquez-Benitez, Uli K. Chettipally, Steven P. Dehmer, Gopikrishna Kunisetty, Rashmi Sharma, Adina S. Rauchwerger, Patrick J. O’Connor, Anupam B. Kharbanda

**Affiliations:** 1 HealthPartners Institute, Minneapolis, Minnesota, US; 2 The Permanente Medical Group, Oakland, California, US; 3 Kaiser Permanente Division of Research, Oakland, California, US; 4 Children’s MN, Department of Pediatric Emergency Medicine, Minneapolis, Minnesota, US

**Keywords:** Clinical Decision Support, Pediatric Emergency Medicine, Appendicitis, Risk Stratification

## Abstract

**Background:**

Appendicitis is a common surgical emergency in children, yet diagnosis can be challenging. An electronic health record (EHR) based, clinical decision support (CDS) system called Appy CDS was designed to help guide management of pediatric patients with acute abdominal pain within the Health Care Systems Research Network (HCSRN).

**Objectives:**

To describe the development and implementation of a clinical decision support tool (Appy CDS) built independently but synergistically at two large HCSRN affiliated health systems using well-established platforms, and to assess the tool’s Triage component, aiming to identify pediatric patients at increased risk for appendicitis.

**Results:**

Despite differences by site in design and implementation, such as the use of alerts, incorporating gestalt, and other workflow variations across sites, using simple screening questions and automated exclusions, both systems were able to identify a population with similar appendicitis rates (11.8 percent and 10.6 percent), where use of the full Appy CDS would be indicated.

**Discussion:**

These 2 HCSRN sites designed Appy CDS to capture a population at risk for appendicitis and deliver CDS to that population while remaining locally relevant and adhering to organizational preferences. Despite different approaches to point-of-care CDS, the sites have identified similar cohorts with nearly identical background rates of appendicitis.

**Next Steps:**

The full Appy CDS tool, providing personalized risk assessment and tailored recommendations, is undergoing evaluation as part of a pragmatic cluster randomized trial aiming to reduce reliance on advanced diagnostic imaging. The novel approaches to CDS we present could serve as the basis for future ED interventions.

## Introduction

Abdominal pain is a common reason for children and adolescents to seek care in the emergency department (ED) [1]. Imaging with ultrasound (US) or computed tomography (CT) has been promoted to improve diagnostic accuracy when evaluating patients with acute abdominal pain [2,3]. In the past 20 years, US and CT use have increased dramatically among patients with suspected appendicitis, especially for children receiving care in general ED settings. Although in some adult cohorts, increased use of diagnostic imaging has been associated with decreases in negative appendectomies, similar improvements in health outcomes among children with acute abdominal pain have not occurred [4,5,6,7]. Potential drawbacks of US include that the procedure is operator dependent, availabilty is inconsistent in the community-setting, and US is prone to indeterminate results. Negative consequences of CT include increased costs, prolonged ED visits, and exposure to ionizing radiation [8,9,10,11,12].

Although appendicitis is the most common surgical emergency in children, its diagnosis remains a challenge. We have developed and validated a pediatric appendicitis risk calculator (pARC), which can accurately predict risk for appendicitis in pediatric patients presenting to the ED [13]. We have emebedded pARC within an electronic health record (EHR)-linked clinical decision support (CDS) system, Appy CDS. Appy CDS is an innovative automated, validated risk prediction tool designed to provide point-of-care CDS to providers for ED pediatric patients with acute abdominal pain. The full Appy CDS uses EHR and web-based algorithms to identify pediatric patients at risk for appendicits, and to provide recommendations regarding next steps in care, consistent with the consensus of clinical experts and informed by a personalized appendicitis risk calculation or pARC score.

A key element in implementing Appy CDS is to first identify a target population where calculation of an appendicitis risk score is likely to impact care. In this paper, we show that using separate strategies and platforms that accommodate variations by health systems, but with similar principles, we were able to identify similar cohorts of patients presenting to the emergency department with abdominal pain and at risk for appendicitis. Along with a detailed description of our intervention, we highlight how this work has benefitted from commonalities across the Health Care Systems Research Network (HCSRN). We also describe adaptations that were needed to accommodate variations by health system in their existing EHR infrastructure, clinical workflow, and preferences of clinical leaders.

## Objectives

### Overview and setting

Appy CDS was developed for use as part of a prospective pragmatic cluster-randomized clinical trial at 17 general EDs in two large HCSRN affilitated care systems. Six sites within HealthPartners (HP) are located in the Upper Midwest of the United States and eleven sites in Kaiser Permanente of Northern California (KPNC) are located across Northern California. These 17 EDs provide care to a diverse pediatric patient population (34 percent Hispanic, 19 percent African-American, and 11 percent Asian). None of the participating EDs are university-based; however, 5 EDs have academic affiliations. Each ED currently uses the EpiCare EHR (Verona, WI). Collectively, the 17 participating EDs have more than 650,000 annual ED visits, of which 101,014 are in patients aged 5–20 years.

### Target population

Appy CDS is designed to provide targeted clinical decision support for patients ages 5–20 years presenting to a participating ED with a chief complaint of abdominal pain. Using data from the EHR and gathered from Triage-based alerts introduced at all participating EDs, we apply automated exclusions, in real-time, for patients with a current pregnancy, trauma, previous abdominal surgery, or one of several chronic conditions, including cystic fibrosis, sickle cell anemia, or inflammatory bowel disease.

### Development of Appy CDS and Triage alert

A primary aim of Appy CDS was to develop a way to identify pediatric patients with acute abdominal pain at increased risk for appendicitis. Having the functionality to accurately identify the target population is a key first step towards targeting the CDS for the “right” patients.

### Workflow Assessment

Contrary to standard workflows and procedures that are typically followed in outpatient settings, a considerable challenge to implementing CDS in the ED was identifying when was the “right” time to introduce decision support in an unpredictable environment with variations in workflow. We therefore conducted detailed, site specific workflow analyses and stakeholder interviews at each site within KPNC and HP to assess the following roles and responsibilities:

1. Who initally assesses patients, entering vital signs, and documenting chief complaint
2. Which providers routinely manage medically stable pediatric patients with accute abdominal pain
3. At what point in care are orders for laboratory work and diagnostic imaging placed
4. At what point in care are surgeons consulted in cases of suspected appendicitis
5. What is the availability of ultrasound and onsite radiology for interpretation of diagnostic studies
6. For patients needing surgery, at what age would transfer to a facility with pediatric surgeons be required

For HP, this first step also involved gathering information about where in the encounter process a Best Practice Advisory (BPA) in the nursing workflow could be introduced.

### Stakeholder Engagement

At the onset of the study, both sites convened independent Clinical Advisory Councils consisting of ED providers, site PIs, Pediatric Radiologists, Pediatric Surgeons as well as IT experts from the participating health systems, who provided input on and approved the Appy CDS system. Stakeholders provided feedback on display features, thresholds for care recommendations for the full CDS, plans for training and monitoring usage, and establishing open communication as future changes in the EHR structure or standards of care could impact the CDS. At HP, nurse manager stakeholders participated in the Clinical Advisory Council and approved of engaging nurses to complete screening questions, as directed through BPAs.

## Description of Appy CDS

**Figure 1:**
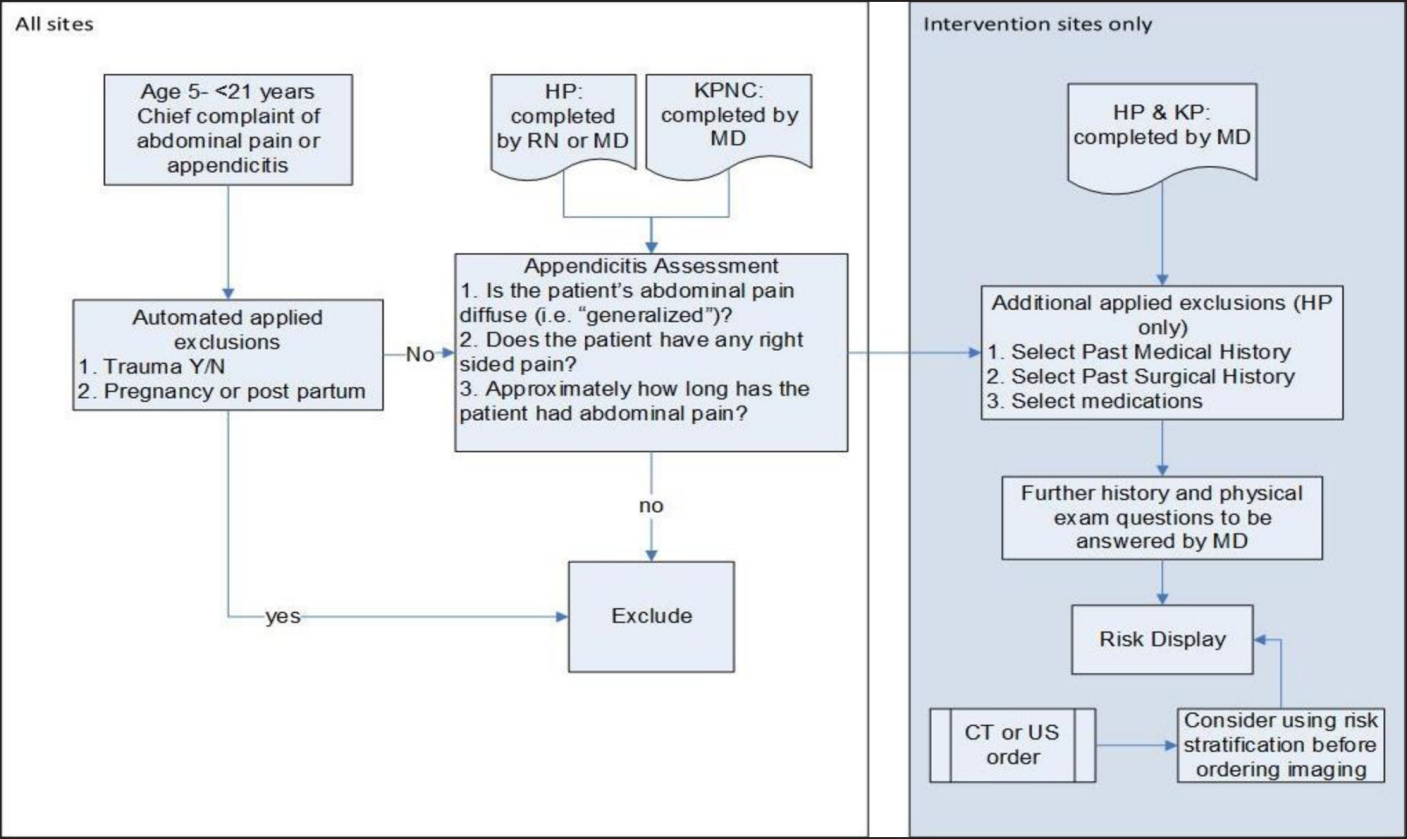
High Level Schematic of Appy CDS System.

The Appy CDS intervention tool is a point-of-care CDS tool consisting of 1) pARC, a validated risk calculator [13,14], 2) a clinical summary, and 3) management suggestions delivered within the EHR via a web-based platform. The tool automatically pulls in prior diagnoses, medications, laboratory, and other clinical data, if available. At intervention sites, the Appy CDS assigns an exact “risk for appendicitis” and provides targeted recommendations regarding management and a summary that can be pasted into the clinical note. Appy CDS was developed concurrently at HP and KPNC, building on existing research platforms within each health system. The tool is consistent across the two health systems but also tailored to local needs with differences in user interface design and workflow. A high level schematic of the design is illustrated in Figure 1. The algorithms underlying the CDS reside on distinct web sites housed on secure servers owned by each health system. Specific details about the intervention design and differences across systems is described in more detail below.

### Appy CDS Design at HealthPartners

Previous outpatient CDS systems implemented in HP have utilized BPAs to notify nurses when a patient meets eligibility criteria for using CDS. This method has proved successful in previous work so we took a similar approach for implementing Appy CDS in the ED [15,16]. With input from ED and Information Systems & Technology leaders, we built the Appy CDS with 3 distinct components: 1) The Triage Alert was designed to be completed as part of nursing workflow, however it triggers for the provider after 10 minutes if questions were not completed during the nursing workflow as a back-up. 2) The MD Alert triggers at intervention sites on provider login to the EHR if the nursing workflow is complete. The alert includes a hyperlink to the full CDS. Finally, 3) an Imaging CDS with link to full CDS, triggers at intervention sites when a provider begins to order a CT or US. Each component is described in detail below.

#### Triage Alert

**Figure 2:**
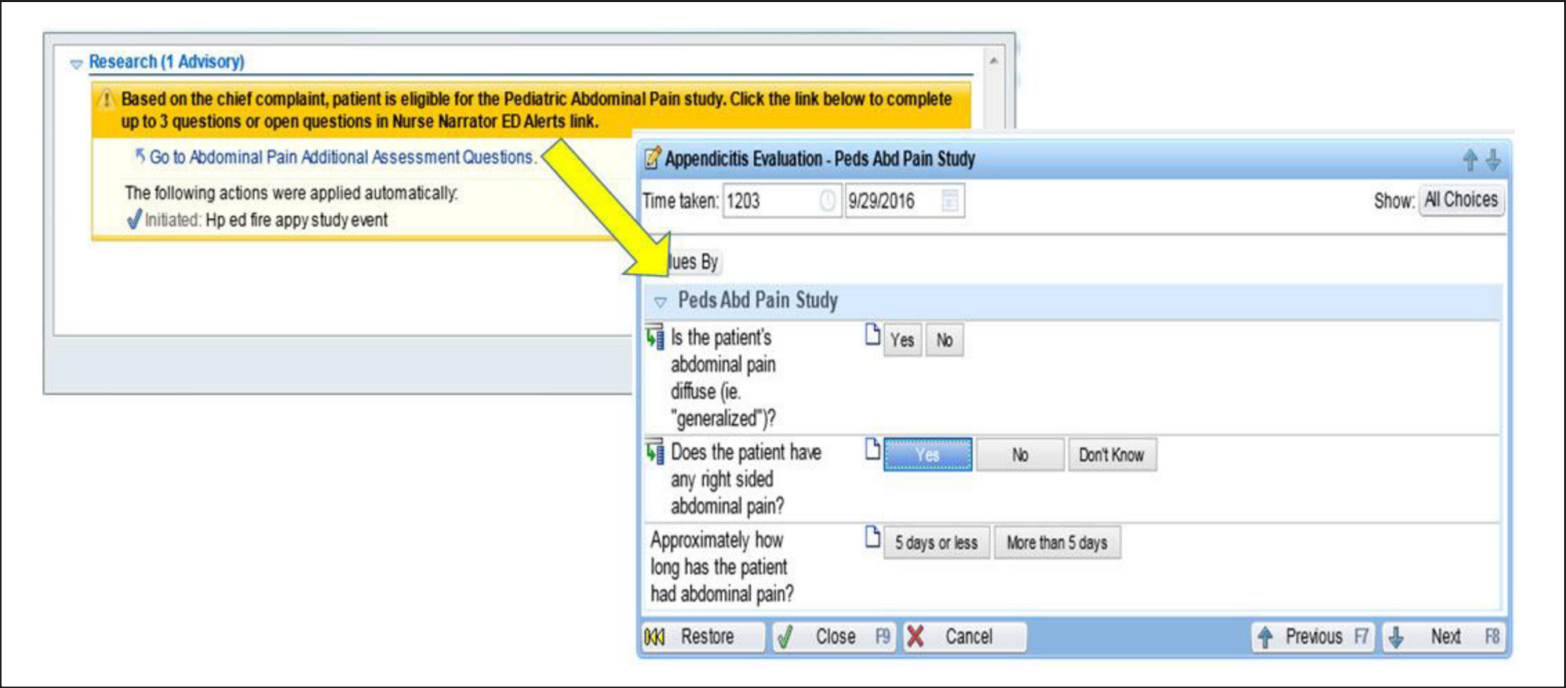
HP Appy CDS BPA with link to eligibility questions.

The Triage alert is a best practice advisory built in the HP EHR that uses automated data to identify potentially eligible study participants. Data variables used to target the study population include age, chief complaint, pregnancy status, and trauma status. If the patient meets the initial study criteria (chief complaint of abdominal pain, age 5–20 years, not pregnant and without trauma), the BPA will display in the ED nursing navigator. The advisory prompts the user to click a link to complete up to 3 questions that further assess eligibility: 1) Is the patient’s abdominal pain diffuse (i.e., generalized), 2) Does the patient have any right-sided abdominal pain, and 3) Approximately how long has the patient had abdominal pain. The Triage alert and associated questions are illustrated in Figure 2. Patients with right-sided or diffuse abdominal pain lasting 5 days or less are enrolled (Figure 2).

#### MD Alert

At intervention sites only, a provider EHR-based alert will trigger for patients enrolled or identified by the *Triage Alert* as at risk for appendicitis. This alert will provide a recommendation that if appendicitis is being considered the provider should “order a complete blood count (CBC) and refer to the risk-stratification algorithm” (accessible via embedded hyperlink). The full CDS includes a collection of additional clinical parameters, such as verifying history and presentation, and results of the CBC with differential (if available).

**Figure 3:**
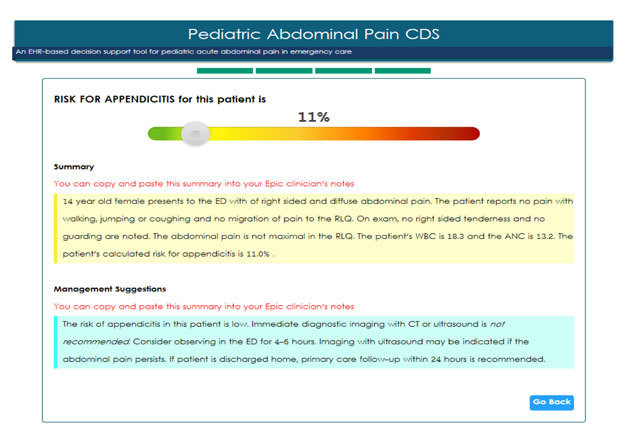
User interface at HPMG of Appy CDS risk prediction showing output, summary, and management suggestions for a fictional patient.

The full Appy CDS prompts providers to verify the trauma and pregnancy status and confirm that the patient has no prior history of abdominal surgery. Next, the CDS prompts the user to confirm the responses to the *Triage Alert* questions that have been pre-populated. Lab results, specifically the white blood cell count (WBC) and neutrophil percent, are also pre-populated if the data are available in the EHR and are used to perform the risk calculation. If lab results are not available at the time the provider is calculating risk, a theoretical value can be entered and used to calculate risk. The option for entering theoretical values was put in place to allow physicians to explore how the risk for appendicitis varies by WBC. However, the preferred workflow is for pARC to be based on actual lab values. There is currently no plan to evaluate pARC results using theoretical values, although this may be considered for secondary analysis. Next, the CDS presents a minimal number of questions on the patient’s presentation and physical exam, that when completed, enables the user to calculate a patient-specific appendicitis risk. Finally, the full CDS provides targeted recommendations regarding management, based on the patient’s appendicitis risk. The summary and management suggestions can be pasted into the clinical note. The user interface of the full Appy CDS at HP is illustrated in Figure 3.

#### Imaging CDS

The final component of the Appy CDS at HP at the intervention sites appears when a provider orders a CT or US for a study-eligible pediatric patient prior to viewing the Appy CDS. The alert prompts the provider to calculate the patient’s appendicitis risk before placing an imaging order.

### Appy CDS Design at Kaiser Permanente

KPNC has a well-established platform for delivering CDS in the ED, called RISTRA, an acronym for RIsk STRAtification. RISTRA is a web services based software application that extracts condition-specific patient data from the EHR, collects information from provider input, uses calculators to risk stratify and displays risk scores and patient-specific management recommendations to the provider in real time at the point of care [17,18]. Providers access RISTRA through a link embedded in the EHR that once clicked, it opens a research portfolio landing page that includes the Appy CDS application. Analogous to HP sites, providers at both intervention and control sites have access to the Triage component via RISTRA that establishes patient eligibility based on age 5–20 years, chief complaint of abdominal pain, and pain less than 5 days as well as applying automated exclusion criteria (not pregnant, no trauma, or prior appendicitis). Similar in format to HP, providers at all KPNC sites are prompted to verify the auto-populated data for accuracy. However, unlike HP, the provider is prompted to enter yes/no for several additional signs or symptoms of appendicitis including duration of pain, nausea or vomiting, anorexia, pain migration or pain with walking, guarding, and location of maximal tenderness on exam.

For providers at KPNC intervention sites, the next step is to assign a *gestalt* assessment of appendicitis risk based on their experience and clinical knowledge using a sliding scale in the Appy CDS. If physician gestalt is less than 10 percent, a risk score will not be calculated, but if ≥10 percent, the full CDS 1) pulls in the WBC, 2) calculates a risk score, 3) provides a patient summary that can be copied into the provider notes. The gestalt element of the CDS is unique to KPNC and provides an opportunity for additional investigation, beyond the overall study goals.

**Figure 4:**
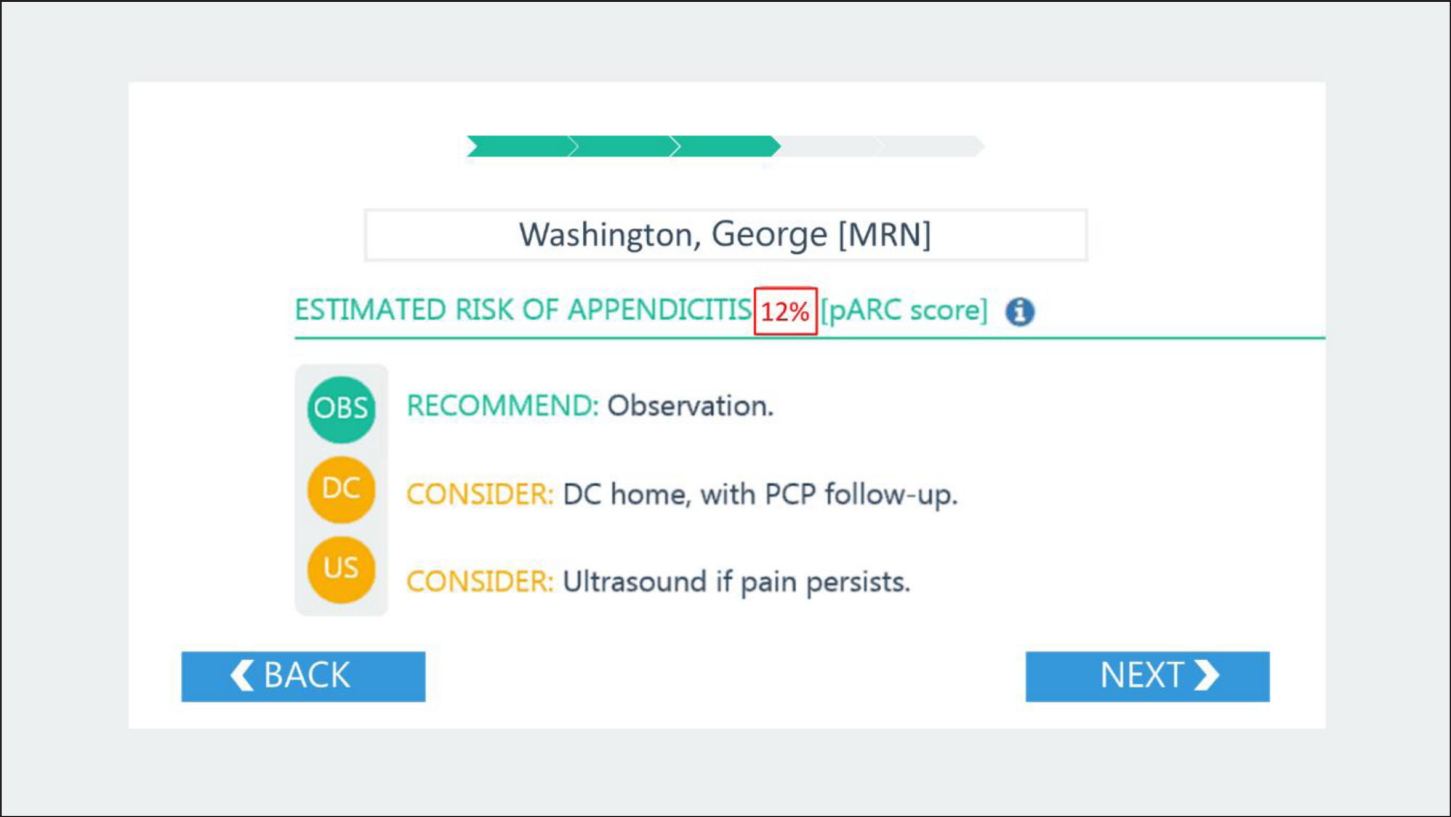
User interface at KPNC of Appy CDS risk prediction output and recommendations for a fictional patient.

Congruent with the tool at HP, RISTRA has mechanisms in place for when the WBC has not been completed and results have not yet returned. In these cases, the provider will see a form with an option to refresh RISTRA once the results are available in the EHR. The provider also has the option to use a sliding scale to visualize how the pARC score might vary by theoretical WBC values. Once the WBC has resulted and the provider refreshes the RISTRA form, the provider can continue with the CDS to obtain the actual calculated risk of appendicitis. An example of the full Appy CDS user interface in RISTRA used at KPNC is shown in Figure 4.

### Implementation, Training, and Feedback

The Appy CDS was rolled out in phases at both sites starting with the Triage phase. As described above, the Triage alert at HP displays to nurses or providers when a patient is potentially eligible based on chief complaint of abdominal pain and age. The alert prompts the nurse or provider to complete 3 questions, which establishes eligibility. At KPNC, providers receive a text alert if a patient presents with abdominal pain and are within the study age range. The text alert notifies the provider that the patient may be eligible and prompts them to activate RISTRA to complete the Triage alert questions [20]. After an 8-month Triage phase, the full Appy CDS tool was rolled out at intervention sites *only*. Table 1 summarizes the different elements of Appy CDS across each system for the Triage phase and the full Appy CDS.

**Table 1:** Elements of the Full Appy CDS Across Systems.

	HP	KPNC

Stakeholder engagement and involvement in design and workflow considerations	+	+
CDS embedded within an existing emergency department CDS platform, RISTRA	–	+
Use of best practice advisories (BPAs) to prompt nurses or providers to complete screening questions in nurse workflow for patients 5–20 years with a chief complaint abdominal pain	+	–
Use of text messages to physicians to prompt to complete screening questions for patients 5–20 years of age with a chief complaint abdominal pain	–	+
Identification and application of exclusions, including pregnancy, current trauma, and prior appendectomy, in real-time	+	+
Use of a web-based algorithm, combining data extracted in real-time from the electronic health record and history and physical exam findings entered by the treating provider to calculate a pediatric appendicitis risk score (pARC)	+	+
Use of physician gestalt to identify patients as low risk and not requiring an appendicitis risk score calculation	–	+
At all participating clinical sites, after applying exclusions and gestalt, collection of data from providers for all eligible patients to calculate risk for appendicitis (pARC)	–	+
At intervention sites only, display of the calculated risk for appendicitis (pARC) and recommendations regarding next steps in care	+	+
Patient summary from CDS can be copied and pasted into the clinical note in the electronic health record	+	+
At intervention sites only, use of BPA to indicate to provider when a patient with completed screening questions is eligible and recommend further data entry to calculate risk for appendicitis (pARC)	+	–
At intervention sites only, use of BPA at time of ordering advanced diagnostic imaging, for eligible patients, and for those with chief complaint abdominal pain and screening questions not completed	+	–
Use of a theoretical white blood cell count to calculate a theoretical appendicitis risk score in patients where blood tests have not been ordered or when results of blood tests are pending	+	+

+ indicates element incorporated into design of CDS system. – indicates element is not incorporated into CDS system.

Methods to improve knowledge and uptake of the CDS have been used throughout the project. Initially, nurses at HP were trained via presentations at staff meetings at all locations followed by one-on-one trainings a few months into the study at the larger sites in effort to reach staff from all shifts. Refresher training has been provided as needed and reminders about the study are periodically presented at staff “huddles”. Providers were also informed of the study at staff meetings. At HP, if Triage alert based questions are completed, names are entered into a monthly drawing for a chance to win a $50 gift card in an effort to boost enrollment and engagment of staff.

To address lack of awareness and lack of familiarity with the CDS system, physician champions were recruited early on at KPNC from each participating ED. Providers at KPNC receive communications about the study and are offered a $5 gift card if they access Appy CDS through RISTRA.

At HP, the study was presented at role-level staff meetings (staff, resident, and physician assisstant (PA) meetings). PA and Resident Champions were recruited at a later date to help promote the tool after an intially slow uptake. Providers at HPMG are also offered a $5 gift card each time they use the full Appy CDS to calculate a risk score. Additionally, leaders from nursing, staff physicians, residents, and PAs receive a summary report on a monthly basis. The reports show enrollment per site for all sites and enrollment plus CDS use for intervention only sites. The project team also provides periodic reports on the performance of the tool in the local population.

## Ethical Considerations

The study is governed by appropriate Data Use Agreements and the Institutional Review Board (IRB) at each institution. We have requested and received waivers of written informed consent from the KPNC and HP IRBs for patients for the following reasons: (a) All options identified by the intervention are evidence-based and have been agreed to as the standard of care by key stakeholders at participating hospitals. Furthermore, no care is advocated that would deviate substantially from the standard of care for pediatric patients with abdominal pain. Therefore, our intervention does not represent any additional risk to patients beyond the routine risk that all patients assume whenever they have contact with the medical care system. (b) At intervention training sessions, we emphasize that it is inappropriate for an ED provider to follow suggested treatment options without further checking the clinical status of a given patient and that they must use our CDS tools as adjuncts, not replacements, for clinical judgment. (c) It would be impractical to consent patients (due to large numbers of patients—up to 10 percent of all pediatric ED visits) and impossible to answer the primary research questions (due to selection effects related to consent) if written informed consent of patients were required. Although unlikely, there is a small chance that implementation of the acute abdominal pain CDS tool will increase the likelihood of a missed case of appendicitis. To answer this question, we monitor for missed cases of appendicitis and rate of perforated appendicitis, along with other important safety outcomes, throughout the study and report missed cases to the Independent Data Monitoring Committee.

## Results of Triage CDS

The purpose of the *Triage Alert* (Triage-based trigger) is to reliably identify a study cohort at elevated risk for appendicitis at both the intervention and control EDs. This cohort comprises the analytic sample that will be used to determine the impact of the full Appy CDS system on CT and US use. In addition, data from *Triage Alert* is included in the appendicitis risk algorithm, as detailed above. Assessment of the *Triage Alert* consists of 2 steps. First, the frequency that the Triage questions are noted in the EHR (pain duration <5 days, pain on right side, generalized or diffuse pain) is identified. Second, among pediatric patients with complete data in the EHR, the negative and positive predictive value of the Triage tool is determined. We hypothesized in our protocol that among pediatric patients with complete data, the Triage tool will yield a positive predictive value above 20 percent and a negative predictive value above 99 percent. Following implementation, we prospectively track the performance of the *Triage Alert* every 6 months at each site and report performance metrics (sensitivity, specificity, positive predictive value, and negative predictive value). Table 2 shows the positive predictive value (PPV) and negative predictive value (NPV) of the Triage component of Appy CDS for patients enrolled October 2017 through September 2018. Despite applying different implementation methods, the PPV of the Triage component is 11.8 percent in HP population and 10.6 percent in KPNC population, consistent across sites. Similarly, the negative predictive value (NPV) of the Triage component overall is 97 at HP and 100 at KPNC (Table 2).

**Table 2:** : Enrollment status distribution, rates of appendicitis, and positive predictive value (PPV) and negative predictive value (NPV) of CDS.

	HealthPartners	Kaiser Permanente

Potential Eligible, n	2389	26346
Enrollment status, n (%)		
Enrolled	1078 (45.1)	3654 (13.9)
Excluded based on triage	627 (26.2)	1862 (7.1)
No response	684 (28.6)	20830 (79.0)
Appendicitis, n (%)		
Enrolled (PPV)	127 (11.8)	386 (10.6)
Excluded based on triage	18 (2.9)	0 (0)
No response	21 (3.1)	762 (3.7)
Not Enrolled (NPV%)*	(97)	(100)

* Negative predictive value is measured only for the triage CDS and does not include those that do not respond.

Table 3 shows baseline characteristics for the enrolled population. Similar gender and age distributions occur across sites although the mean age in the HP population is 14.01 years, slightly higher than 11.46 years in the KPNC population. This may, in part, be due to the transferring of younger patients to nearby pediatric hospitals. Not surprising, each health care system provides services to different demographic communities, which is reflected in the race and ethnic population, with higher rates of Latino in California (37.6 percent) versus in Minnesota (10.4 percent).

**Table 3:** Characteristics of Enrolled Population.

	HealthPartners (N = 1078)	Kaiser Permanente (N = 3654)

Female, n (%)	657 (61.0)	1961 (53.7)
Mean age (SD) in years	14.0 (4.6)	11.5 (4.2)
*Race, n (%)*		
Asian	60 (5.6)	554 (15.2)
Black or African American	157 (14.6)	393 (10.8)
White	696 (64.6)	2318 (63.5)
Other or unknown	165 (15.3)	389 (10.6)
*Ethnicity, n (%)*		
Hispanic or Latino	112 (10.4)	1373 (37.6)

## Discussion

This pragmatic trial is ongoing at two large health care systems in the HCSRN which each have histories of successfully developing, implementing, and evaluating CDS systems. At the onset of this collaboration, each organization sought to remain consistent with and build on existing work and technology infrastructure. The existing systems were of benefit to the project as the fundamental steps were already in place and no extra time was needed to build the tool from scratch. Initial project team discussions revolved around how to retain the integrity of the individual systems while meeting the needs of the overall study. The similarities across the systems are that they 1) use automated methods to identify potentially eligible children age 5–20 years with a chief complaint of abdominal pain, 2) present an alert to users via text alerts or BPAs that prompts for further evaluation, 3) calculate a patient-specific pARC score using minimal EHR data passed to a web service, and, at intervention sites, 4) display the risk for appendicitis, a summary of presentation, and management suggestions appropriate for the level of risk of the individual patient.

The implementation strategy was carefully planned to incorporate advice of practicing providers and an overall desire to fit efficiently within existing workflows. ED providers work in hectic environments and are apt to forget to use the system, despite best intensions. As is the case with many pragmatic trials, elements of implementation were adapted and modified over the study intervention period to improve use in the targeted population.

Beyond efforts to capture a cohort with similar baseline risk, in large pragmatic trials such as this the reality is that local factors can affect implementation and uptake of new tools. This needs to be considered in multisite studies even when leveraging a shared data model. At HP, we built a series of EHR BPAs designed for nurses to complete to identify patient eligibility. This approach has worked well in outpatient settings with standard rooming processes. However, the ED is less predictable and has wide variation in workflow, especially at larger sites where these variations may affect enrollment. In contrast, KPNC relies on providers to complete the Triage questions and uses a text message system to remind them of potentially eligible patients. The RISTRA approach has fewer ‘moving parts’ but there is risk of providers forgetting to use it if not reminded with prompts in the EHR, or via text or other communications.

Despite the challenges, this project has benefitted greatly from the experience that both HCSRN sites brought to the study team. The collaborative approach to problem solving may have been difficult to achieve with organizations outside of the network. The biggest research challenge has been finding the balance between achieving target enrollment and enrolling the appropriate target population. At the onset of the study, we hypothesized that the target population for pARC calculation should have an appendicitis rate of 20 percent. However, in practice, this would have decreased our sample size, and likely we would have excluded patients at risk of appendicitis where a risk score calculation would be indicated. The Triage Alert has thus far been successful in excluding few patients with appendicitis.

## Next steps

Effectiveness of the full Appy CDS system on the reliance of CT and US will be evaluated in this cluster-randomized trial, with results reported at the end of the intervention period. Evaluation of study outcomes are planned for late 2019. Until then, we will continue to monitor the CDS and provide feedback to providers to help mitigate barriers to use such as alert fatigue or other barriers, as well as continue to monitor effectiveness of the Appy CDS and safety outcomes. Once the evaluation is complete, it is up to individual medical groups to approve further dissemination activities in their organizations. From a technical standpoint, the tools can be easily activated at all sites within both systems.

If successful, this sophisticated web-based EHR-linked CDS system could be adapted and implemented broadly in a range of acute care settings to both standardize and personalize care delivered to pediatric patients. Alternative approaches to risk score calculation could also be considered in low resource settings through mobile devices. Additionally, given the pragmatic variations in implementation, this study will provide a unique opportunity to evaluate optimal strategies for future dissemination.

## References

[1] Pitts, SR. National Hospital Ambulatory Medical Care Survey: 2006 Emergency Department Summary; 2006. 18958996

[2] Rao, PM, Rhea, JT, Novelline, RA, Mostafavi, AA, Lawrason, JN, McCabe, CJ and Helical, CT. combined with contrast material administered only through the colon for imaging of suspected appendicitis. AJR Am J Roentgenol. 1997; 169(5): 1275–1280. DOI: 10.2214/ajr.169.5.9353441 9353441

[3] Garcia Pena, BM, Mandl, KD, Kraus, SJ, et al. Ultrasonography and limited computed tomography in the diagnosis and management of appendicitis in children. JAMA. 1999; 282(11): 1041–1046. DOI: 10.1001/jama.282.11.1041 10493202

[4] Krajewski, S, Brown, J, Phang, PT, Raval, M and Brown, CJ. Impact of computed tomography of the abdomen on clinical outcomes in patients with acute right lower quadrant pain: A meta-analysis. Can J Surg. 2011; 54(1): 43–53. DOI: 10.1503/cjs.023509 21251432 PMC3038359

[5] Bachur, RG, Hennelly, K, Callahan, MJ, Chen, C and Monuteaux, MC. Diagnostic imaging and negative appendectomy rates in children: Effects of age and gender. Pediatrics. 2012; 129(5): 877–884. DOI: 10.1542/peds.2011-3375 22508920

[6] Partrick, DA, Janik, JE, Janik, JS, Bensard, DD and Karrer, FM. Increased CT scan utilization does not improve the diagnostic accuracy of appendicitis in children. J Pediatr Surg. 2003; 38(5): 659–662. DOI: 10.1016/jpsu.2003.5017 12720164

[7] Martin, AE, Vollman, D, Adler, B and Caniano, DA. CT scans may not reduce the negative appendectomy rate in children. J Pediatr Surg. 2004; 39(6): 886–890, discussion 886–890. DOI: 10.1016/j.jpedsurg.2004.02.034 15185219

[8] Lee, SL, Walsh, AJ and Ho, HS. Computed tomography and ultrasonography do not improve and may delay the diagnosis and treatment of acute appendicitis. Arch Surg. 2001; 136(5): 556–562. DOI: 10.1001/archsurg.136.5.556 11343547

[9] Miglioretti, DL, Johnson, E, Williams, A, et al. The use of computed tomography in pediatrics and the associated radiation exposure and estimated cancer risk. JAMA Pediatr. 2013; 167(8): 700–707. DOI: 10.1001/jamapediatrics.2013.311 23754213 PMC3936795

[10] Karakas, SP, Guelfguat, M, Leonidas, JC, Springer, S and Singh, SP. Acute appendicitis in children: Comparison of clinical diagnosis with ultrasound and CT imaging. Pediatr Radiol. 2000; 30(2): 94–98. DOI: 10.1007/s002470050023 10663520

[11] Pritchett, CV, Levinsky, NC, Ha, YP, Dembe, AE and Steinberg, SM. Management of acute appendicitis: The impact of CT scanning on the bottom line. J Am Coll Surg. 2010; 210(5): 699–705, 705–697. 20421033 10.1016/j.jamcollsurg.2009.12.043

[12] Lin, KH, Leung, WS, Wang, CP and Chen, WK. Cost analysis of management in acute appendicitis with CT scanning under a hospital global budgeting scheme. Emerg Med J. 2008; 25(3): 149–152. DOI: 10.1136/emj.2007.050963 18299362

[13] Kharbanda, AB, Vazquez-Benitez, G, Ballard, DW, et al. Development and Validation of a Novel Pediatric Appendicitis Risk Calculator (pARC). Pediatrics. 2018; 141(4). DOI: 10.1542/peds.2017-2699 PMC5869337 29535251

[14] Murray, DM, Varnell, SP and Blitstein, JL. Design and analysis of group-randomized trials: A review of recent methodological developments. Am J Public Health. 2004; 94(3): 423–432. DOI: 10.2105/AJPH.94.3.423 14998806 PMC1448268

[15] O’Connor, PJ, Sperl-Hillen, JM and Rush, WA, et al. Impact of electronic health record clinical decision support on diabetes care: A randomized trial. Ann Fam Med. 2011; 9(1): 12–21. DOI: 10.1370/afm.1196 21242556 PMC3022040

[16] Kharbanda, EO, Asche, SE, Sinaiko, AR, et al. Clinical Decision Support for Recognition and Management of Hypertension: A Randomized Trial. Pediatrics. 2018; 141(2). DOI: 10.1542/peds.2017-2954 PMC5810603 29371241

[17] Simon, LE, Rauchwerger, AS, Chettipally, UK, et al. Real-time Text Message Alerts to Emergency Physicians Identifying Potential Study Candidates Increases Clinical Trial Enrollment. Paper presented at: HCSRN Annual Conference 2018; Mpls, MN. 10.1093/jamia/ocz118 PMC6798557 31340023

[18] Zeger, SL and Liang, KY. Longitudinal data analysis for discrete and continuous outcomes. Biometrics. 1986; 42(1): 121–130. DOI: 10.2307/2531248 3719049

